# Impact of different hysterectomy techniques on postoperative clinical and sexual outcomes: A retrospective analysis in a tertiary center

**DOI:** 10.1371/journal.pone.0353020

**Published:** 2026-07-20

**Authors:** Yusuf Başkıran, Gamze Karababa, Kazım Uçkan, İzzet Çeleğen

**Affiliations:** 1 Department of Obstetrics and Gynecology, Istinye University Faculty of Medicine, Istanbul, Türkiye; 2 Department of Obstetrics and Gynecology, Silvan Dr Yusuf Azizoğlu State Hospital, Diyarbakır, Türkiye; 3 Department of Obstetrics and Gynecology, Faculty of Medicine, Van Yuzuncu Yil University, Van, Türkiye; 4 Department of Public Health, Faculty of Medicine, Van Yuzuncu Yil University, Van, Türkiye; Xiangya Hospital Central South University, CHINA

## Abstract

**Background:**

Hysterectomy is a commonly performed gynecologic procedure; however, evidence regarding how different surgical techniques affect postoperative vaginal anatomy and sexual function remains inconsistent. This study aimed to evaluate postoperative vaginal length, female sexual function, male partner sexual function, and perioperative outcomes following total abdominal hysterectomy (TAH), total laparoscopic hysterectomy (TLH), and total vaginal hysterectomy (TVH) performed for benign gynecological indications.

**Methods:**

In this retrospective cohort study, 466 women who underwent total hysterectomy between January 2020 and June 2023 at a tertiary referral center were included. Patients were grouped according to surgical technique: TAH, TLH, or TVH. Vaginal length measurements, Female Sexual Function Index (FSFI) scores, perioperative laboratory values, blood transfusion requirement, and postoperative complications were analyzed. Male partner sexual function was assessed using the International Index of Erectile Function-5 (IIEF-5) in a subset of patients. Multivariable linear and logistic regression analyses were performed to identify factors associated with postoperative outcomes. Estimated intraoperative blood loss was not consistently available in the retrospective database and could not be included in the transfusion model.

**Findings:**

Of the 466 patients, 124 underwent TAH, 177 TLH, and 165 TVH. Preoperative vaginal length and FSFI scores were comparable across groups. Postoperative vaginal length differed significantly according to surgical technique, with the greatest reduction observed in the TLH group (p < 0.001). Postoperative female FSFI scores did not differ significantly among groups (p > 0.05). Among male partners with available data, IIEF-5 scores showed a statistically significant postoperative decline (mean ± SD: 14.87 ± 3.31 preoperatively vs. 14.10 ± 2.23 postoperatively; p < 0.001); however, this partner-related finding was exploratory. Blood transfusion was required in 12.0% of patients (95% CI, 9.4–15.3), and postoperative complications occurred in 8.2% (95% CI, 6.0–11.0). In multivariable analyses, lower preoperative hemoglobin levels were associated with a higher likelihood of blood transfusion. Longer operative time was associated with postoperative complications (OR per minute = 1.02; 95% CI, 1.01–1.03; p < 0.001), likely reflecting surgical complexity rather than a direct causal effect.

**Conclusions:**

Hysterectomy technique was associated with differences in postoperative vaginal length, but these anatomical differences did not translate into significant differences in early postoperative female sexual function. Lower preoperative hemoglobin was associated with blood transfusion requirement, although the lack of estimated intraoperative blood loss data limits interpretation of transfusion-related findings. Longer operative time was associated with postoperative complications and may reflect greater surgical complexity. The observed decline in male partner IIEF-5 scores should be interpreted cautiously as an exploratory finding, and future prospective studies incorporating both patient- and partner-reported outcomes with longer follow-up are warranted.

## Introduction

Hysterectomy is the most commonly performed pelvic surgical procedure in gynecology [1]. While the most frequent indication is uterine leiomyoma, other benign or malignant gynecological conditions such as abnormal uterine bleeding, adenomyosis, endometriosis, malignancies, and pelvic organ prolapse may also necessitate hysterectomy [2].

Today, hysterectomy can be performed using several surgical approaches, including abdominal, vaginal, laparoscopic, robotic-assisted, and transvaginal natural orifice transluminal endoscopic surgery techniques [3,4]. The selection of surgical route is influenced by multiple patient-, disease-, surgeon-, and institution-related factors, including the indication for surgery, uterine size, pelvic anatomy, presence of prolapse, previous surgery, comorbidities, patient preference, available technology, and surgeon experience [5]. Therefore, comparisons among hysterectomy techniques in retrospective observational studies should be interpreted with caution because surgical approach is not randomly assigned and may reflect underlying differences in case complexity and patient selection.

Laparoscopic hysterectomy has gained widespread acceptance as a minimally invasive alternative to abdominal and vaginal approaches. It is associated with several well-documented advantages over abdominal surgery, including shorter hospital stay, reduced postoperative pain, faster return to normal activities, lower incidence of wound infections, and decreased blood loss [6,7]. Although comparisons between abdominal and minimally invasive methods are often complicated by differences in patient selection, uterine size, surgical indication, and surgical expertise, current evidence generally suggests higher complication rates in open abdominal approaches [8]. Consequently, the use of laparoscopic techniques has significantly increased, especially in cases where vaginal hysterectomy is not feasible [7,9,10]. In parallel, robotic-assisted hysterectomy has increasingly been adopted in contemporary gynecologic practice; however, its use remains dependent on institutional resources, cost, availability of robotic platforms, and surgeon training. As robotic hysterectomy was not routinely performed at our center during the study period, it was not included in the present analysis.

Beyond perioperative outcomes, postoperative anatomical and functional outcomes are important components of recovery after hysterectomy. Vaginal length may differ according to surgical technique, cuff closure, extent of vaginal tissue excision, and indication for surgery. However, the relationship between postoperative vaginal anatomy and sexual function remains complex. Sexual function after hysterectomy may be influenced not only by anatomical changes, but also by preoperative symptoms, relief of bleeding or pain, hormonal status, psychological factors, relationship dynamics, and sociocultural context. Therefore, anatomical differences between surgical techniques may not necessarily translate into clinically meaningful differences in postoperative sexual function.

In this study, we aimed to retrospectively analyze total hysterectomy procedures performed for benign indications over a three-and-a-half-year period at a tertiary care center. While previous studies have largely focused on perioperative parameters such as blood loss, complications, and hospital stay, fewer studies have evaluated postoperative vaginal length together with female and partner sexual function across different hysterectomy techniques. We further examined demographic and clinical predictors of transfusion and complications, thereby providing a comprehensive assessment that bridges surgical outcomes with functional and quality-of-life measures. Given the retrospective design and non-random allocation of surgical approach, the findings should be interpreted as associations rather than evidence of causal superiority of one surgical technique over another.

## Materials and methods

### Study design and setting

This retrospective observational cohort study was conducted at the Department of Obstetrics and Gynecology, Van Training and Research Hospital, a tertiary referral center in eastern Türkiye. The study period spanned from January 2020 to June 2023. The study included patients who underwent total hysterectomy for benign gynecological indications using one of the three conventional surgical approaches routinely performed at our institution during the study period: total abdominal hysterectomy, total laparoscopic hysterectomy, or total vaginal hysterectomy. Robotic-assisted hysterectomy was not routinely performed at our center during the study period and was therefore not included in the analysis. The study was reported in accordance with the Strengthening the Reporting of Observational Studies in Epidemiology (STROBE) guidelines.

### Participants

The study included women aged 18–75 years who underwent total hysterectomy for benign gynecological indications during the study period. Surgical approach was determined in routine clinical practice according to surgical indication, uterine size, pelvic anatomy, presence of prolapse, previous surgery, comorbidities, surgeon judgment, and institutional practice, rather than by random allocation. Patients were excluded if they had incomplete demographic or clinical records, inadequate compliance with clinical examination, missing sexual function assessment data, or insufficient perioperative documentation for the outcomes of interest. Patients undergoing hysterectomy for malignant indications, obstetric indications, or emergency life-threatening hemorrhage were not included. After application of these criteria, 466 patients were included in the final analysis.

### Variables and outcomes

Patient-level data included demographic characteristics age, height, weight, body mass index, gravida, parity, comorbidities, and history of previous surgery; surgical variables including indication score, uterine size, operative time, and type of hysterectomy; and clinical outcomes including preoperative and postoperative hemoglobin and hematocrit levels, blood transfusion requirement, postoperative complications, vaginal length, and sexual function scores. Estimated intraoperative blood loss was not consistently recorded in the retrospective surgical records and therefore could not be included as a study variable or incorporated into the transfusion models.

Postoperative complications were defined as any adverse medical or surgical event occurring within 30 days after hysterectomy, including bleeding requiring reoperation; wound-related complications such as infection, hematoma, or dehiscence; febrile morbidity defined as a body temperature ≥38°C on two or more consecutive postoperative days excluding the first 24 hours; urinary tract infection or urinary tract injury confirmed by laboratory, imaging, or operative findings; and readmission or reintervention related to postoperative events. Minor and self-limiting conditions such as transient nausea, mild ileus, or short-term fever were not classified as complications.

The primary outcome was the change in vaginal length from preoperative to postoperative assessment. Secondary outcomes included postoperative female sexual function assessed by the Female Sexual Function Index (FSFI), male partner sexual function assessed by the International Index of Erectile Function-5 (IIEF-5), postoperative complication rates, and blood transfusion requirement. Analyses of male partner sexual function were considered exploratory because partner data were available for a subset of 297 cases. Analyses involving partner outcomes were restricted to these patients, and missing partner data were excluded by listwise deletion.

### Data sources and measurement

Clinical and perioperative data were extracted retrospectively from electronic hospital records. Operative time was defined as the interval from skin incision to completion of the surgical procedure. All hysterectomies were performed by gynecologic surgeons at a tertiary referral center according to standard institutional surgical practice. The choice of surgical route was based on clinical indication, pelvic anatomy, uterine size when available, presence of prolapse, previous surgical history, comorbidities, and surgeon judgment.

Vaginal length was measured intraoperatively using a hysterometer immediately before hysterectomy and at the completion of the procedure, ensuring a standardized assessment across surgical techniques. Hemoglobin and hematocrit levels were obtained from routine laboratory tests performed preoperatively and during the early postoperative period as part of standard clinical care. Estimated intraoperative blood loss was not consistently documented in the electronic surgical records and therefore could not be analyzed.

Female and male sexual function were evaluated using validated instruments, namely the Female Sexual Function Index and the International Index of Erectile Function-5, respectively [11,12], based on documented preoperative and postoperative assessments recorded in patient files. Postoperative sexual function assessments were conducted at routine follow-up visits, typically around 8 weeks after surgery, with an acceptable window of 6–12 weeks. When multiple postoperative assessments were available, the measurement closest to 8 weeks was used for analysis. Therefore, sexual function outcomes in this study should be interpreted as early postoperative functional assessments rather than long-term sexual function outcomes.

The study data were accessed for research purposes between 01/05/2023 and 15/06/2023 in accordance with institutional regulations.

### Study size

The final sample size reflected all eligible patients who met the inclusion criteria during the study period. No a priori sample size calculation was performed because of the retrospective design. Therefore, the analyses were exploratory and based on the available cohort.

### Ethics approval

The study protocol was approved by the Non-Interventional Clinical Research Ethics Committee of Van Regional Training and Research Hospital approval date: 26/04/2023; protocol no: 2023/09–04. The study was conducted in accordance with the Declaration of Helsinki. Due to the retrospective design and the use of de-identified routinely collected clinical data, the requirement for informed consent was waived by the ethics committee. The minimal anonymized dataset underlying this study is restricted due to patient privacy and institutional regulations. Qualified researchers may request access to the data by contacting the Non-Interventional Clinical Research Ethics Committee of Van Regional Training and Research Hospital (Contact Email: vaneah@saglik.gov.tr, Phone: + 90 432 222 00 10). The study data were accessed and extracted for research purposes after ethics approval, between 01/05/2023 and 15/06/2023, in accordance with institutional regulations. Clinical trial registration was not applicable because this was a retrospective observational study.

### Statistical analysis

Statistical analyses were performed using IBM SPSS Statistics version 20.0 IBM Corp., Armonk, NY, USA. The distribution of continuous variables was assessed visually using histograms and Q–Q plots and analytically using the Kolmogorov–Smirnov test. Continuous variables were summarized as mean ± standard deviation or median interquartile range, as appropriate, while categorical variables were presented as number and percentage.

Comparisons among surgical groups were performed using one-way analysis of variance with Tukey’s post-hoc test for normally distributed continuous variables. Paired sample t-tests were used for within-group preoperative and postoperative comparisons. Categorical variables were compared using Pearson’s chi-square test or Fisher’s exact test, as appropriate.

Multivariable analyses included linear regression models for postoperative vaginal length and postoperative FSFI scores, and binary logistic regression models for postoperative complications and blood transfusion requirement. Candidate variables were selected based on clinical relevance and availability in the retrospective database. For postoperative vaginal length, the model included preoperative vaginal length, surgical type, age, body mass index, uterine size, and operative time. For postoperative FSFI score, the model included preoperative FSFI score, surgical type, age, body mass index, and postoperative complications. For blood transfusion, the model included operative time, preoperative hemoglobin, and preoperative hematocrit. For postoperative complications, the model included age, body mass index, operative time, and uterine size.

Because preoperative hemoglobin and hematocrit are biologically related parameters, multicollinearity was assessed using variance inflation factors before interpretation of the transfusion model. Estimated intraoperative blood loss could not be included in the transfusion model because it was not consistently available in the retrospective surgical records. Uterine size was included in models when available; missing uterine size data were not imputed. Analyses involving male partner IIEF-5 scores were restricted to patients with available partner data and were considered exploratory.

Model diagnostics included assessment of multicollinearity using variance inflation factors, goodness-of-fit using Nagelkerke R² for logistic regression models, and calibration using the Hosmer–Lemeshow test. All analyses were exploratory in nature. A two-sided p-value <0.05 was considered statistically significant, without adjustment for multiple comparisons.

## Results

Descriptive characteristics of the study population are summarized in Table 1. A total of 466 patients were included in the analysis. The mean age was 48.74 ± 7.20 years range, 31–75. Mean height and weight were 159.32 ± 5.82 cm and 78.13 ± 13.80 kg, corresponding to a mean body mass index BMI of 30.43 ± 5.38 kg/m². The mean numbers of pregnancies and births were 4.40 ± 2.41 and 3.32 ± 2.03, respectively. Comorbid conditions were present in 64.8% of patients, and 77.5% had a history of previous surgery. The mean hysterectomy indication score was 9.33 ± 2.77. Uterine size measurements were available for 244 patients 52.4%, with a mean uterine size of 9.96 ± 2.19 cm, while uterine size was not recorded in 222 patients 47.6%. Missing uterine size values were not imputed.

**Table 1:** Descriptive Characteristics of the Study Population.

Variable	N	Mean ± SD	Min–Max
**Age (years)**	466	48.74 ± 7.20	31–75
**Height (cm)**	466	159.32 ± 5.82	145–178
**Weight (kg)**	466	78.13 ± 13.80	48–126
**Body mass index (kg/m²)**	466	30.43 ± 5.38	17.9–49.2
**Gravida**	466	4.40 ± 2.41	0–15
**Parity**	466	3.32 ± 2.03	0–15
**Indication score**	466	9.33 ± 2.77	1–16
Uterine size (cm)*	244	9.96 ± 2.19	8–18
**Operative time (min)**	466	151.71 ± 42.19	60–300
**Preoperative hemoglobin (g/dL)**	466	12.22 ± 1.79	6–16
**Postoperative hemoglobin (g/dL)**	466	10.62 ± 1.80	5–15
**Preoperative hematocrit (%)**	466	38.06 ± 4.78	20–48
**Postoperative hematocrit (%)**	466	33.15 ± 4.61	16–46
**Preoperative vaginal length (cm)**	466	8.52 ± 0.75	8–10
**Postoperative vaginal length (cm)**	466	7.86 ± 0.79	7–10
**Preoperative FSFI score**	466	23.52 ± 4.46	12–31
**Postoperative FSFI score**	466	23.64 ± 3.59	17–32
**Partner preoperative IIEF-5 score**	297	14.87 ± 3.31	11–22
**Partner postoperative IIEF-5 score**	297	14.10 ± 2.23	11–22

Abbreviations: SD = standard deviation; BMI = body mass index; FSFI = Female Sexual Function Index; IIEF-5 = International Index of Erectile Function-5.

*Uterine size data were available for 244 patients.

The mean operative time was 151.71 ± 42.19 minutes. Mean preoperative hemoglobin and hematocrit levels were 12.22 ± 1.79 g/dL and 38.06 ± 4.78%, respectively, and decreased postoperatively to 10.62 ± 1.80 g/dL and 33.15 ± 4.61%. Blood transfusion was required in 12.0% of patients 95% CI, 9.4–15.3, and postoperative complications occurred in 8.2% 95% CI, 6.0–11.0. Mean vaginal length decreased from 8.52 ± 0.75 cm preoperatively 95% CI, 8.45–8.59 to 7.86 ± 0.79 cm postoperatively 95% CI, 7.79–7.93. Female sexual function, assessed using the Female Sexual Function Index FSFI, showed minimal overall change from preoperative to postoperative evaluation 23.52 ± 4.46 vs. 23.64 ± 3.59. Among male partners with available data n = 297, mean International Index of Erectile Function-5 IIEF-5 scores declined from 14.87 ± 3.31 preoperatively to 14.10 ± 2.23 postoperatively; this partner-related finding was considered exploratory because partner data were available only for a subset of the cohort.

Changes in vaginal length and female sexual function according to hysterectomy technique are presented in Table 2. Patients were grouped as total abdominal hysterectomy TAH, n = 124, total laparoscopic hysterectomy TLH, n = 177, and total vaginal hysterectomy TVH, n = 165. Preoperative vaginal length and preoperative FSFI scores were comparable among the groups p = 0.954 and p = 0.995, respectively. Postoperative vaginal length differed significantly according to surgical technique, with the shortest postoperative vaginal length observed in the TLH group p < 0.001. The greatest reduction in vaginal length occurred following TLH −1.08 ± 0.64 cm, followed by TAH −0.49 ± 0.61 cm, whereas a modest increase in vaginal length was observed after TVH + 0.34 ± 0.58 cm. Postoperative FSFI scores did not differ significantly among the groups p = 0.508, and changes in FSFI scores ΔFSFI were also similar across surgical techniques p = 0.267. Male partner IIEF-5 scores showed a statistically significant postoperative decline across surgical groups p < 0.001; however, this result should be interpreted as an exploratory partner-related outcome rather than as a definitive indicator of clinically meaningful deterioration.

**Table 2:** Changes in Vaginal Length and Female Sexual Function According to Hysterectomy Technique.

Variable	TAH (n = 124)	TLH (n = 177)	TVH (n = 165)	p-value
**Preoperative vaginal length (cm)**	8.53 ± 0.76	8.53 ± 0.77	8.51 ± 0.73	0.954
**Postoperative vaginal length (cm)**	8.04 ± 0.77ᵃ	7.45 ± 0.67ᵇ	8.17 ± 0.74ᵃ	<0.001
**Δ Vaginal length (cm)**	−0.49 ± 0.61	−1.08 ± 0.64	+0.34 ± 0.58	<0.001
**Preoperative FSFI score**	23.54 ± 4.49	23.49 ± 4.45	23.52 ± 4.48	0.995
**Postoperative FSFI score**	23.56 ± 3.59	23.46 ± 3.65	23.90 ± 3.52	0.508
**Δ FSFI score**	+0.02 ± 2.11	−0.03 ± 2.18	+0.38 ± 2.04	0.267

Data are presented as mean ± standard deviation. One-way ANOVA was used for between-group comparisons. Superscript letters indicate statistically significant differences between groups based on Tukey’s HSD post-hoc test (p < 0.05). Δ indicates postoperative minus preoperative values.

Variables associated with postoperative complications in univariate analyses are shown in Table 3. When patients with complications n = 38 were compared with those without complications n = 428, no statistically significant differences were observed in age, BMI, uterine size, operative time, or postoperative vaginal length in univariate comparisons all p > 0.05. The distribution of hysterectomy techniques was also similar between patients with and without postoperative complications p = 0.980. Complication rates were 8.1% for TAH, 7.9% for TLH, and 8.5% for TVH. These univariate findings suggest that postoperative complications were not clearly associated with surgical route in crude comparisons.

**Table 3:** Variables Associated with the Development of Postoperative Complications.

Variable	No complication (n = 428)	Complication (n = 38)	p-value
**Age (years)**	48.69 ± 7.26	49.37 ± 5.45	0.365
**BMI (kg/m²)**	30.44 ± 5.36	29.95 ± 5.15	0.876
**Surgical type**			0.980
**– TAH (n = 124)**	114 (91.9%)	10 (8.1%)	
**– TLH (n = 177)**	163 (92.1%)	14 (7.9%)	
**– TVH (n = 165)**	151 (91.5%)	14 (8.5%)	
**Uterine size (cm)**	10.40 ± 2.87	9.92 ± 2.12	0.236
**Operative time (min)**	151.91 ± 41.82	148.00 ± 49.84	0.653
**Postoperative vaginal length (cm)**	7.86 ± 0.79	7.79 ± 0.82	0.897

Data are presented as mean ± SD or n (%). Independent samples t-test was used for continuous variables; chi-square or Fisher’s exact test for categorical variables. TAH = Total Abdominal Hysterectomy; TLH = Total Laparoscopic Hysterectomy; TVH = Total Vaginal Hysterectomy; BMI = Body Mass Index.

Multivariable linear regression analysis identifying factors associated with postoperative vaginal length is presented in Table 4. The model was statistically significant p < 0.001. Preoperative vaginal length was the strongest predictor of postoperative vaginal length B = 0.742; 95% CI, 0.69–0.80; p < 0.001. After adjustment for age, BMI, uterine size, operative time, and preoperative vaginal length, surgical technique remained associated with postoperative vaginal length. Compared with TAH, TLH was associated with a shorter postoperative vaginal length B = −0.506; 95% CI, −0.66 to −0.36; p < 0.001, whereas TVH was associated with a longer postoperative vaginal length B = 0.268; 95% CI, 0.03–0.51; p = 0.029. Age, BMI, uterine size, and operative time were not significantly associated with postoperative vaginal length in this model. Because uterine size data were not available for all patients, findings involving this covariate should be interpreted with caution.

**Table 4:** Factors Affecting Postoperative Vaginal Length.

Variable	B coefficient (95% CI)	p-value	Interpretation
**Preoperative vaginal length**	0.742 (0.69–0.80)	<0.001	Strongest positive predictor
**Surgical type (TLH vs TAH)**	−0.506 (−0.66 to −0.36)	<0.001	Shorter vaginal length in TLH
**Surgical type (TVH vs TAH)**	0.268 (0.03–0.51)	0.029	Longer vaginal length in TVH
**Age**	0.002 (−0.005 to 0.007)	0.634	Not significant
**BMI**	0.006 (−0.003 to 0.015)	0.181	Not significant
**Uterine size**	0.012 (−0.006 to 0.030)	0.180	Not significant
**Operative time**	0.0001 (−0.001 to 0.001)	0.787	Not significant

Data were analyzed using multivariate linear regression. Dependent variable: postoperative vaginal length. Independent variables: age, BMI, surgical type, uterine size, operative time, and preoperative vaginal length. TAH = Total Abdominal Hysterectomy; TLH = Total Laparoscopic Hysterectomy; TVH = Total Vaginal Hysterectomy; BMI = Body Mass Index. Statistically significant results are shown in bold (p < 0.05).

Factors associated with postoperative female sexual function are summarized in Table 5. The multivariable linear regression model was statistically significant p < 0.001. Preoperative FSFI score was the only significant predictor of postoperative FSFI score B = 0.671; 95% CI, 0.63–0.71; p < 0.001. After adjustment for age, BMI, surgical type, postoperative complications, and preoperative FSFI score, hysterectomy technique was not significantly associated with postoperative FSFI score. Age, BMI, and postoperative complications were also not significantly associated with postoperative FSFI outcomes in this model. These findings suggest that baseline female sexual function was more strongly related to early postoperative FSFI scores than surgical route in the present cohort.

**Table 5:** Factors Associated with Postoperative FSFI Score.

Variable	B coefficient (95% CI)	p-value	Interpretation
**Preoperative FSFI score**	0.671 (0.63–0.71)	**<0.001**	Strongest positive predictor
**Surgical type (TLH vs TAH)**	−0.080 (−0.538 to 0.378)	0.732	Not significant
**Surgical type (TVH vs TAH)**	0.378 (−0.099 to 0.855)	0.120	Not significant
**Postoperative complication (yes)**	0.245 (−0.414 to 0.904)	0.466	Not significant
**Age**	−0.004 (−0.029 to 0.022)	0.781	Not significant
**BMI**	−0.007 (−0.043 to 0.028)	0.683	Not significant

Data were analyzed using multivariate linear regression. Dependent variable: postoperative Female Sexual Function Index (FSFI) score. Independent variables: age, BMI, surgical type, presence of postoperative complication, and preoperative FSFI score. TAH = Total Abdominal Hysterectomy; TLH = Total Laparoscopic Hysterectomy; TVH = Total Vaginal Hysterectomy; BMI = Body Mass Index. Statistically significant results are shown in bold (p < 0.05).

Multivariable logistic regression analysis of factors associated with blood transfusion is presented in Table 6. After adjustment for operative time, preoperative hemoglobin, and preoperative hematocrit levels, lower preoperative hemoglobin was significantly associated with a higher likelihood of blood transfusion (OR = 0.39; 95% CI, 0.26–0.60; p < 0.001). Operative time showed a borderline association with transfusion requirement (p = 0.055), whereas preoperative hematocrit was not significantly associated with transfusion in this model. Because preoperative hemoglobin and hematocrit are closely related hematologic parameters, the nonsignificant result for hematocrit should be interpreted in the context of adjustment for hemoglobin. Estimated intraoperative blood loss was not available in the retrospective database and therefore could not be included in the transfusion model.

**Table 6:** Multivariate Logistic Regression Analysis of Factors Associated with Blood Transfusion.

Variable	OR (95% CI)	p-value	Interpretation
**Operative time (min)**	1.01 (1.00–1.01)	0.055	Borderline association
**Preoperative hemoglobin (g/dL)**	0.39 (0.26–0.60)	<0.001	Protective factor
**Preoperative hematocrit (%)**	1.10 (0.94–1.27)	0.232	Not significant

Multivariate binary logistic regression with blood transfusion (yes/no) as the dependent variable. OR = odds ratio; CI = confidence interval.

Multivariable logistic regression analysis examining factors associated with postoperative complications is shown in Table 7. Longer operative time was significantly associated with a higher likelihood of postoperative complications in the multivariable model (OR per minute = 1.02; 95% CI, 1.01–1.03; p < 0.001). Age, BMI, and uterine size were not significantly associated with postoperative complications. Given the observational design, operative time should be interpreted as a marker that may reflect surgical complexity or intraoperative difficulty rather than as a direct causal factor.

**Table 7:** Multivariate Logistic Regression Analysis of Factors Associated with Postoperative Complications.

Variable	OR (95% CI)	p-value
**Age (years)**	1.02 (0.98–1.07)	0.370
**Body mass index (kg/m²)**	1.02 (0.96–1.08)	0.596
**Operative time (min)**	1.02 (1.01–1.03)	<0.001
**Uterine size (cm)**	1.00 (0.94–1.07)	0.990

Multivariate binary logistic regression with postoperative complication (yes/no) as the dependent variable. OR = odds ratio; CI = confidence interval.

## Discussion

Laparoscopic hysterectomy has become an integral component of contemporary gynecologic surgery as a minimally invasive alternative to the abdominal route. When performed by experienced surgeons, it is consistently associated with shorter hospital stay, reduced postoperative pain, faster recovery, and lower rates of wound-related morbidity compared with open surgery [6,7,13]. These advantages have driven a global shift toward minimally invasive approaches, with surveillance and institutional practice studies showing increasing adoption of laparoscopic hysterectomy and the influence of surgeon expertise on minimally invasive hysterectomy rates [14,15]. This trend is also strongly supported by current international guidelines recommending laparoscopy whenever vaginal hysterectomy is not feasible for benign indications [16,17]. Large registry-based analyses and systematic reviews further indicate that laparoscopic and vaginal hysterectomy yield complication rates that are comparable to, or lower than, those observed with abdominal hysterectomy, despite differences in case complexity and patient selection [4,6,7,18]. In parallel, robotic-assisted hysterectomy has increasingly been incorporated into contemporary gynecologic practice, particularly in centers with access to robotic platforms and specialized training. However, robotic surgery was not routinely performed at our institution during the study period; therefore, the present analysis was limited to abdominal, laparoscopic, and vaginal hysterectomy. This should be considered when interpreting the applicability of our findings to centers where robotic hysterectomy is widely available.

In the present study, postoperative complication rates did not differ significantly among hysterectomy techniques in univariate comparisons. In multivariable analysis including age, body mass index, operative time, and uterine size, operative time was the only variable significantly associated with postoperative complications. This association should not be interpreted as evidence that longer operative duration directly causes complications. Rather, operative time may serve as a surrogate marker of greater surgical complexity, technical difficulty, adhesions, difficult pelvic anatomy, or intraoperative challenges. Similar findings have been reported in large-scale observational studies, demonstrating comparable overall morbidity profiles across hysterectomy techniques when procedures are performed in high-volume or tertiary-care settings [18–23]. Although laparoscopic hysterectomy is often associated with longer operative times, particularly during the learning phase, accumulating evidence suggests that procedure-related risks decrease substantially with increasing surgeon experience and institutional expertise [19–25]. Our findings therefore support the view that laparoscopic hysterectomy can provide perioperative safety comparable to other established techniques in appropriately selected patients; however, because the surgical route was not randomly assigned, this result should be interpreted in the context of possible selection bias and differences in case complexity.

Procedure-specific complications should also be interpreted in relation to intraoperative technical factors. Bowel injury and urinary tract injury are recognized complications of hysterectomy, particularly in patients with endometriosis, previous abdominal surgery, dense adhesions, or difficult pelvic anatomy [26,27]. Conversion to laparotomy may represent an appropriate safety strategy when minimally invasive completion is not feasible [28], and previous laparoscopic hysterectomy series have reported variable rates of intraoperative and postoperative complications depending on case complexity and surgical experience [29]. Contemporary data also suggest that minimally invasive approaches may reduce wound-related morbidity and surgical site infection compared with open abdominal hysterectomy [30]. In addition, standardized perioperative infection-prevention bundles have been associated with reductions in hysterectomy-related surgical site infections, underscoring the importance of institutional protocols in postoperative outcomes [31,32]. In the present retrospective dataset, however, detailed information on specific injury subtypes, adhesions, conversion, and infection-prevention bundle adherence was not systematically available; therefore, these factors could not be analyzed separately.

Blood transfusion represents a clinically relevant perioperative outcome reflecting both patient-related factors and intraoperative conditions. In the present cohort, lower preoperative hemoglobin was significantly associated with a higher likelihood of blood transfusion, whereas preoperative hematocrit was not significant after adjustment for hemoglobin and operative time. This finding is clinically plausible because hemoglobin is a direct parameter used in perioperative transfusion decision-making. In addition, hemoglobin and hematocrit are closely related hematologic measures, and the nonsignificant result for hematocrit may partly reflect shared information between these variables within the same model. In line with previous reports, transfusion may be influenced by baseline hematologic status, surgical complexity, uterine size, operative conditions, and perioperative management strategies [33]. However, estimated intraoperative blood loss was not consistently available in the retrospective surgical records and therefore could not be incorporated into the transfusion model. Similarly, the database did not contain a separate variable defining emergency surgery or acute bleeding status. Although the study was restricted to hysterectomies performed for benign gynecologic indications, unmeasured differences in bleeding-related indications or surgical urgency may have influenced transfusion risk. Therefore, the transfusion findings should be interpreted as reflecting associations within the available retrospective dataset rather than a complete explanatory model of perioperative blood loss.

Functional outcomes, particularly vaginal length and sexual function, represent important yet often underemphasized dimensions of postoperative recovery after hysterectomy. Postoperative sexual function is multifactorial and may be influenced by pelvic floor function, preoperative symptoms, relief of pain or bleeding, hormonal status, body image, psychological adaptation, partner relationship dynamics, and sociocultural context [34]. In our cohort, postoperative vaginal length decreased overall, with the greatest reduction observed following laparoscopic hysterectomy and the longest postoperative vaginal length observed after vaginal hysterectomy. These findings are consistent with previous comparative studies demonstrating technique-specific differences in postoperative vaginal anatomy and sexual function after hysterectomy [35,36]. Nevertheless, despite these anatomical differences, female sexual function scores remained largely unchanged after surgery and did not differ significantly among hysterectomy techniques. This observation reinforces the concept that postoperative sexual function is not solely determined by vaginal length or surgical anatomy [34–37]. Preoperative symptoms, relief of pain or bleeding, hormonal status, body image, psychological adaptation, partner relationship dynamics, and sociocultural context may all contribute to postoperative sexual function.

All procedures included in the present study were total hysterectomies. Although the potential impact of cervical preservation on sexual outcomes remains a topic of debate, recent randomized trials, prospective cohort studies, and systematic reviews have consistently demonstrated no clinically meaningful differences in sexual function between total and supracervical hysterectomy [38–40]. These data suggest that symptom relief, hormonal milieu, partner relationship dynamics, and psychosocial factors may exert a greater influence on postoperative sexual satisfaction than cervix preservation alone. However, the sexual function assessments in the present study were conducted relatively early in the postoperative period, typically around 8 weeks after surgery within an acceptable 6–12-week window. Therefore, our findings should be interpreted as early postoperative functional outcomes. Longer-term changes in sexual function, including adaptation after complete healing and resumption of regular sexual activity, could not be evaluated in this retrospective dataset.

Male partner sexual function, assessed using the IIEF-5, showed a statistically significant postoperative decline across surgical groups. This finding is interesting because partner-related outcomes after hysterectomy have been less frequently evaluated than patient-reported female sexual function. However, the magnitude and clinical significance of this decline should be interpreted cautiously. Partner data were available only for a subset of patients, and the analysis was exploratory rather than a primary endpoint. In addition, IIEF-5 scores may be influenced by age, comorbidities, psychological factors, relationship dynamics, and sociocultural perceptions surrounding gynecologic surgery, many of which could not be fully controlled in the present retrospective design. While our finding differs from some previous reports describing neutral or favorable partner outcomes following hysterectomy [37], it should be viewed as hypothesis-generating. Future prospective studies with longer follow-up and more detailed partner-level variables are needed to clarify whether hysterectomy has a measurable and clinically meaningful effect on male partner sexual function.

### Limitations

This study has several limitations that should be considered when interpreting the findings. First, the retrospective, single-center design limits causal inference and may reduce generalizability to settings with different patient populations, surgical expertise, and access to minimally invasive or robotic surgery. Second, the surgical approach was not randomly assigned but was determined by clinical indication, uterine size, pelvic anatomy, presence of prolapse, previous surgery, comorbidities, surgeon judgment, and institutional practice. Therefore, selection bias and residual confounding by case complexity cannot be excluded. Third, estimated intraoperative blood loss was not consistently recorded, and separate data on emergency surgery or acute bleeding status were not available; these missing variables limit the interpretation of transfusion-related findings. Fourth, uterine size measurements were missing for a substantial proportion of patients, which may have reduced the precision of analyses involving this variable and limited adjustment for surgical complexity. Fifth, sexual function outcomes were assessed using self-reported instruments and relatively early after surgery, which may not capture longer-term recovery, adaptation, or changes in sexual activity. Finally, male partner sexual function data were available only for a subset of participants, potentially introducing selection bias and limiting the representativeness of partner-related findings. Operative time, identified as a factor associated with postoperative complications, should also be interpreted as a possible marker of surgical complexity rather than a direct causal mechanism.

Despite these limitations, the study is strengthened by its relatively large sample size, inclusion of multiple conventional hysterectomy techniques, and comprehensive evaluation of perioperative, anatomical, and functional outcomes. The concurrent assessment of female and male partner sexual function provides a broader perspective on the potential functional impact of hysterectomy, although the partner-related findings should be regarded as exploratory.

## Conclusion

In this retrospective cohort study, hysterectomy technique was associated with postoperative vaginal length, with the greatest reduction observed after laparoscopic hysterectomy and the greatest preservation observed after vaginal hysterectomy. However, these anatomical differences did not translate into significant differences in early postoperative female sexual function, supporting the view that postoperative sexual health is multifactorial and not determined by vaginal length alone.

Lower preoperative hemoglobin was associated with a higher likelihood of blood transfusion, whereas operative time was associated with postoperative complications, likely reflecting surgical complexity rather than a direct causal effect of procedure duration. Because estimated intraoperative blood loss and emergency or acute bleeding status were not consistently available, the transfusion findings should be interpreted cautiously.

Male partner IIEF-5 scores showed a statistically significant postoperative decline; however, this partner-related result was exploratory, based on a subset of patients, and its clinical significance remains uncertain. Future prospective, multicenter studies with longer follow-up, detailed surgical complexity variables, estimated blood loss data, and both patient- and partner-reported outcomes are needed to better clarify the anatomical, functional, and couple-centered effects of different hysterectomy techniques.

## Supporting information

S1 Data Minimal anonymized dataset used in the analysis. This file contains the anonymized dataset supporting the main analyses of the study. (XLSX)
